# Modeling Magnetomyograms of Uterine Contractions during Pregnancy Using a Multiscale Forward Electromagnetic Approach

**DOI:** 10.1371/journal.pone.0152421

**Published:** 2016-03-28

**Authors:** Mengxue Zhang, Vanessa Tidwell, Patricio S. La Rosa, James D. Wilson, Hari Eswaran, Arye Nehorai

**Affiliations:** 1 Department of Electrical and Systems Engineering, Washington University in Saint Louis, Saint Louis, Missouri, United States of America; 2 Research & Development/Technology Pipeline Solutions, Monsanto Company, Saint Louis, Missouri, United States of America; 3 Joint Undergraduate Engineering Program - University of Missouri and Washington University in Saint Louis, Saint Louis, Missouri, United States of America; 4 Graduate Institute of Technology, University of Arkansas at Little Rock, Little Rock, Arkansas, United States of America; 5 Department of Obstetrics and Gynecology, University of Arkansas for Medical Sciences, Little Rock, Arkansas, United States of America; PreTel Inc, UNITED STATES

## Abstract

Understanding the mechanisms of uterine contractions during pregnancy is especially important in predicting the onset of labor and thus in forecasting preterm deliveries. Preterm birth can cause serious health problems in newborns, as well as large financial burdens to society. Various techniques such as electromyography (EMG) and magnetomyography (MMG) have been developed to quantify uterine contractions. However, no widely accepted method to predict labor based on electromagnetic measurement is available. Therefore, developing a biophysical model of EMG and MMG could help better understand uterine contractions, interpret real measurements, and detect labor. In this work, we propose a multiscale realistic model of uterine contractions during pregnancy. At the cellular level, building on bifurcation theory, we apply generalized FitzHugh-Nagumo (FHN) equations that produces both plateau-type and bursting-type action potentials. At the tissue level, we introduce a random fiber orientation model applicable to an arbitrary uterine shape. We also develop an analytical expression for the propagation speed of transmembrane potential. At the organ level, a realistic volume conductor geometry model is provided based on magnetic resonance images of a pregnant woman. To simulate the measurements from the SQUID Array for Reproductive Assessment (SARA) device, we propose a sensor array model. Our model is able to reproduce the characteristics of action potentials. Additionally, we investigate the sensitivity of MMG to model configuration aspects such as volume geometry, fiber orientation, and pacemaker location. Our numerical results show that fiber orientation and pacemaker location are the key aspects that greatly affect the MMG as measured by the SARA device. We conclude that sphere is appropriate as an approximation of the volume geometry. The initial step towards validating the model against real MMG measurement is also presented. Our results show that the model is flexible to mimic the limited-propagation magnetic signature during the emergence and decay of a uterine contraction.

## Introduction

The clinical importance of modeling contractions of pregnant uterus lies in better understanding the mechanisms of normal and preterm births. Preterm birth, which occurs before 37 weeks of gestation, has potential to result in serious health problems to preterm infants. The earlier infants are born, the greater their risk of morbidity and mortality. In addition to health problems, preterm birth causes huge financial costs to families and society [1]. As a result, it is urgent to understand the mechanisms under which uterine contractions lead to preterm birth.

The uterus provides a safe environment for developing fetus, which is later expelled through intense contractions. These contractions are primarily regulated by the uterine electrical activities [2, 3]. Previous models [4, 5] rarely consider the physiological properties of myometrium. A computer model consisting of discrete contractile elements that propagate electrical impulses and generate tension is proposed in [4]. The authors predict contraction waveforms by defining contracting and refractory periods. In [5], the authors demonstrate that two mechanisms, action potential propagation and calcium wave propagation, contribute to intercellular communication.

The models presented in [6–11] take into account the physiological aspect of uterine contractions. A simple electrophysiological model of smooth muscle cell is developed in [6]. The limitation of this model is that it only includes sodium and potassium currents, but not the involved physiological ionic currents. In [7], the authors propose a model that considers the electrical and mechanical properties of smooth muscle cells; however the model disregards the time dependency of calcium current. A model is developed to study the process of myometrial excitation and contraction in [8]. This model describes L-type Ca^2+^ current, Ca^2+^ pumps, and Na^+^/Ca^2+^ exchangers without including the time-dependency dynamics. In [9], the authors construct a model in terms of voltage-dependent Na^+^ currents, voltage-dependent Ca^2+^ currents, voltage and calcium-dependent K^+^ currents, a leakage current, and dynamics of intracellular calcium concentration. In [10], the authors provide a more detailed smooth muscle cell model, which has 13 ionic currents and calcium dynamics, to reproduce different types of action potentials. In [11], KCNQ and hERG channel currents are added into the uterine cell model which is developed in [10], enabling the simulations of long-lasting bursting-type action potentials.

Recently, the models focus on characterizing the electrophysiological property of uterine contractions jointly at the cellular, tissue, and organ levels. A 2D multiscale model for uterine electrical activity is presented in [12, 13]. This model considers K^+^ current and the dynamics of Ca^2+^ in the cell scale, applies a 2D isotropic grid of square cells in the tissue scale, and simulates the surface electrohysterogram in the organ scale. In our previous work [14], a multiscale electromagnetic model of uterine contractions is developed. This model is the first to apply 3D multiscale approach to uterine modeling. However, it regards the uterus as a sphere with fixed-angle conductivity tensor, which is an oversimplified version of the real geometry.

In this work, we develop a more realistic multiscale forward model of uterine contractions during pregnancy based on the simplified model in [14]. We first introduce an ionic current model capable of generating both plateau-type and bursting-type action potentials in each myocyte. Specifically, using bifurcation analysis, we develop a generalization of the FitzHugh-Nagumo (FHN) equations capable of generating both types of action potentials. Second, we introduce a random fiber orientation model applicable to an arbitrary uterine shape. An analytical expression for the speed of propagation is also developed. Third, instead of a spherical model, we introduce a realistic model for the volume conductor geometry based on magnetic resonance images (MRI) of a pregnant woman. We also propose a sensor array model matching the SARA device to simulate the real recordings [15–17]. The numerical results illustrate the main characteristics of both types of action potentials. We also demonstrate that the pattern of magnetic field depends greatly on the fiber orientation and pacemaker location, rather than the volume geometry. The spherical volume geometry is therefore a good approximation of uterine geometry to investigate the MMG measurements of the SARA device. We also validate that our modeling approach is flexible to mimic the limited-propagation magnetic field pattern of real patient data during the emergence and decay of contraction.

Our aim is to better understand the uterine structure and propagation of contractions from electrophysiological point of view and to determine which aspects of model configuration have a major influence on the pattern of abdomen-surface magnetic field as measured by the SARA device. By creating a variety of model configuration aspects, e.g., by spatially varying the fiber layout or conductivity properties of the myometrium, and then simulating the magnetic field on abdominal surface for each configuration, we wish to determine the extent to which each aspect of the configuration has influence on the pattern of magnetomyograms. Creating a realistic multiscale forward model of uterine contractions will allow us to better interpret the data of MMG measurements and, therefore, shed light on the prediction of preterm labor.

## Methods

Below, we first review our preliminary work and then elaborate on our improvement to the approach.

### Preliminary work

In the following section, we summarize our preliminary work. In our recent paper [14], we proposed a multiscale forward electromagnetic model of human myometrial contractions during pregnancy, of which the details are listed in Table 1.

**Table 1:** Equations of uterine model in [14].

Magnetic field	$\nabla \;\times \;B\left(r,t\right)=\mu_{0}\left(J_{s}\left(r,t\right)-G\left(r\right)\nabla \phi \left(r,t\right)\right)$ (1)
Electrical potential	$\nabla \cdot G_{A}(r)\nabla \phi (r,t)=0,in\;A$ (2)
	$$\nabla \;\cdot \;G_{U}(r)\nabla \phi (r,t)=0,in\;U$$ (3)
	$$\nabla \;\cdot \;G_{e}^{\prime }\nabla \phi_{\text{e}}(r,t)=-\nabla \cdot \frac{\zeta }{\zeta +1}G_{e}^{\prime }\nabla v_{\text{m}}(r,t),in\;ℳ$$ (4)
Source current density	$J_{s}\left(r,t\right)=-\zeta G_{e}^{{}^{\prime }}\nabla v_{m}\left(r,t\right),in\;M$ (5)
Transmembrane potential model	$\nabla \cdot \frac{\zeta }{\zeta +1}G_{e}^{{}^{\prime }}\nabla v_{m}\left(r,t\right)=a_{m}\left(c_{m}\frac{\partial v_{m}\left(r,t\right)}{\partial t}+J_{\text{ion}}\left(r,t\right)-J_{\text{stim}}\left(r,t\right)\right),in\;M$ (6)
Pacemaker activity model	$J_{\text{stim}}\left(r,t\right)=\frac{1}{\varepsilon_{1}}\sum_{i=1}^{N_{p}}\nu_{i}h_{i}\left(r,t\right)$ (7)
Boundary conditions	$\phi_{\text{e}}(r,t)=\phi_{A}(r,t),in\;\partial ℳ$ (8)
	$${\hat{n}}_{ℳ}\cdot (G_{i}^{\prime }\nabla \phi_{\text{i}}(r,t)+G_{e}^{\prime }\nabla \phi_{\text{e}}(r,t))={\hat{n}}_{ℳ}\;\cdot \;G_{A}\nabla \phi_{A}(r,t),in\;\partial ℳ$$ (9)
	$${\hat{n}}_{ℳ}\cdot G_{i}^{\prime }\nabla v_{\text{m}}(r,t)=0,\;in\;\partial ℳ$$ (10)
	$$\phi_{\text{e}}(r,t)=\phi_{U}(r,t),in\;\partial U$$ (11)
	$${\hat{n}}_{U}\cdot (G_{\text{i}}^{\prime }\nabla \phi_{\text{i}}(r,t)+G_{e}^{\prime }\nabla \phi_{\text{e}}(r,t))={\hat{n}}_{U}\;\cdot \;G_{U}\nabla \phi_{U}(r,t),in\;\partial U$$ (12)
	$${\hat{n}}_{U}\;\cdot \;G_{i}^{\prime }\nabla v_{\text{m}}(r,t)=0,in\;\partial U$$ (13)
	$${\hat{n}}_{A}\;\cdot \;G_{A}\nabla \phi_{A}(r,t)=0,in\;\partial A$$ (14)
	$${\hat{n}}_{ℱ}\;\cdot \;G_{U}\nabla \phi_{U}(r,t)=\lambda ({\hat{n}}_{ℱ}\;\cdot \;G_{ℱ}\nabla \phi_{ℱ}(r,t)),\;in\;\partial ℱ$$ (15)

Our modeling approach was to solve the forward electromagnetic problem of uterine contractions using a four-compartment volume conductor geometry, namely, we computed at the abdominal surface the magnetic field, ***B***(***r***, *t*) Eq (1), and the electrical potential, *ϕ*(***r***, *t*) Eqs (2)–(4), generated by the myometrial current source density, ***J***_s_(***r***, *t*) Eq (5). According to Ohm’s law, the current source density ***J***_s_(***r***, *t*) was defined as the gradient of transmembrane potential, *v*_m_(***r***, *t*) Eq (6). The transmembrane potential *v*_m_(***r***, *t*), using reaction diffusion equations, was modeled as a function of ionic current dynamics, ***J***_ion_(***r***, *t*), stimulus current due to pacemakers, ***J***_stim_(***r***, *t*) Eq (7), anisotropic conductivity, $G_{i}^{{}^{\prime }}$ and $G_{e}^{{}^{\prime }}$, and the corresponding boundary conditions Eqs (8)–(15). As illustrated in [14], in the volume conductor geometry model, A represents the abdominal cavity and ∂A the boundary surface defined by the abdomen. M represents the myometrium, and ∂M and ∂U are its external and internal boundary surfaces, respectively. The volume denoted by U represents the space filled with the amniotic fluid that exists between the internal uterine wall ∂U and the boundary ∂F defined by the fetus volume F. Focusing on plateau-type action potential, we applied the modified version of FitzHugh-Nagumo (FHN) equations [18, 19] to model the ionic currents, ***J***_ion_(***r***, *t*), in each myocyte. We also proposed a general approach to design conductivity tensors, $G_{i}^{{}^{\prime }}$ and $G_{e}^{{}^{\prime }}$, simply assuming that the main axis of the fibers runs vertically from the fundus to the cervix for any uterine shape. To model the volume conductor geometry, we defined a spherical uterus and also a spherical abdomen as a simplification of the real anatomical structure.

Although our preliminary work provides a novel approach to model the contractions in pregnant uterus, some aspects of the model are oversimplified when compared with the realistic case. In the following sections, we will explain explicitly how we develop a more realistic multiscale model taking into consideration the electrophysiological and anatomical characteristics of uterus.

### Bursting-type action potential model based on bifurcation theory

**Fig 1:**
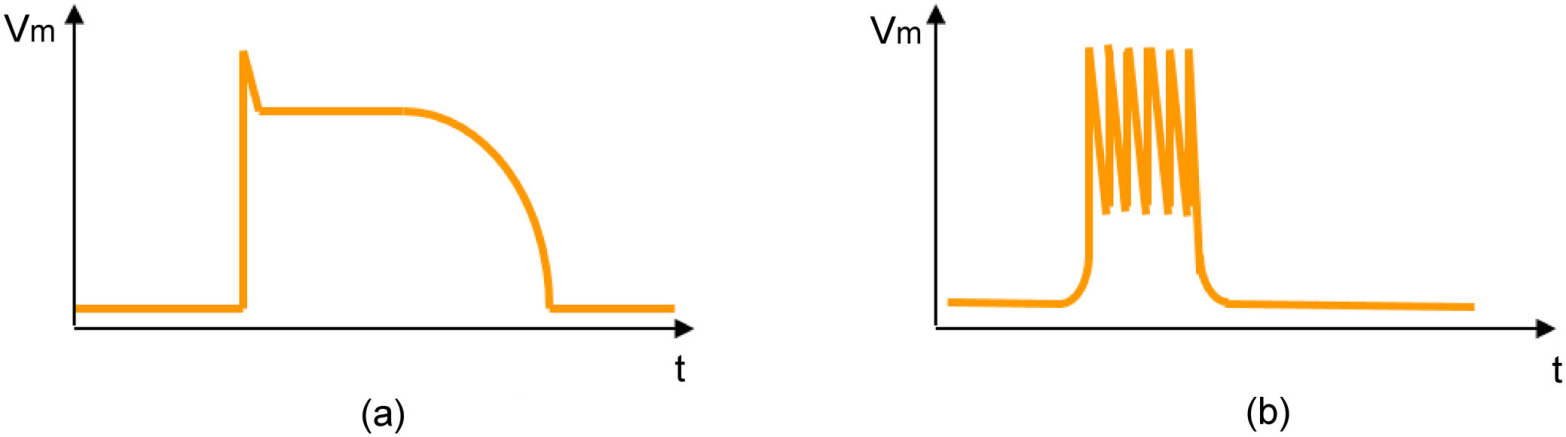
Caricature of the two types of action potentials observed in the human uterus. (a) Plateau-type action potential. (b) Bursting-type action potential.

In this section, we present an ionic current model capable of generating plateau-type and bursting-type action potentials (Fig 1), since both of them are very common in pregnant human uterus [20, 21]. Particularly, we apply a variation of the FHN equations [14], which is given by the following nonlinear dynamical system of ordinary differential equations:

$$\frac{\partial v_{m}}{\partial t}=\frac{1}{\varepsilon_{1}c_{m}}\left(k\left(v_{m}-v_{1}\right)\left(v_{2}-v_{m}\right)\left(v_{m}-v_{3}\right)-w+\nu \right),$$ (16)

$$\frac{\partial w}{\partial t}=\varepsilon_{2}\left(\beta v_{m}-\gamma w+\delta \right),$$ (17)

where *v*_m_ is the action potential, *w* is a state variable, *ν* is the stimulus current amplitude due to pacemaker activity, which is defined in Eq (7), and *ε*_1_, *ε*_2_, *k*, *β*, *γ*, *δ*, *v*_1_, *v*_2_, and *v*_3_ are model parameters.

The behavior of a nonlinear dynamical system depends greatly on the values of its parameters. For example, for certain set of parameters, the FHN model has a stable but excitable equilibrium. That is, if the system undergoes a sufficiently large perturbation, there is a large excursion of variables in phase space before returning to the equilibrium, hence generating plateau-type action potential. There is also a range of values where the FHN model displays limit cycle characteristics; that is, the variables settle on a closed trajectory in phase space, hence spike trains occur in the system.

In this work, we focus on investigating the bursting-type action potential, which occurs when the FHN model displays limit cycle behavior. In order to identify the set of parameters that generates a limit cycle, we apply bifurcation analysis to the variation of the FHN model. Bifurcation analysis is a powerful tool to investigate such properties of nonlinear dynamical systems. It helps to identify the set of parameters with which certain behavior occurs in the systems. The parameter that is varied is known as bifurcation parameter. We assume in this work that the uterus is formed by the same type of cells. The pacemaker activity is initiated for a specified stimulus current but the parameters of the ionic current cell model are exactly the same throughout the uterus. Therefore, the only degree of freedom that we have is the stimulus amplitude, which can be varied at initiation, so it can be regarded as the bifurcation parameter. In other words, our claim is that the cell response changes on the basis of the pacemaker activity.

In bifurcation analysis, equilibrium, limit cycle, and their stabilities appear as a function of the bifurcation parameter. Since the right hand side of Eq (16) is a cubic polynomial of *v*_m_, it is difficult to write explicitly the equilibrium $v_{m}^{*}$ as a function of the bifurcation parameter *ν*. Instead, we rewrite the stimulus amplitude *ν* as a function of the equilibrium $v_{m}^{*}$:

$$\nu \left(v_{m}^{*}\right)=-k\left(v_{m}^{*}-v_{1}\right)\left(v_{2}-v_{m}^{*}\right)\left(v_{m}^{*}-v_{3}\right)+\left(\beta v_{m}^{*}+\delta \right)/\gamma ,$$ (18)

where $v_{m}^{*}$ is the value of action potential at the equilibrium. Therefore, by finding the conditions in which the system has an unstable equilibrium and limit cycle, we can then derive the corresponding values for the stimulus amplitude using Eq (18). In this work, we are interested in the case when there is always only one equilibrium in the FHN model whatever the stimulus amplitude *ν*, for which the parameters should satisfy the following condition (see S1 Appendix):

$$\Delta_{1}≜{\left(v_{1}+v_{2}+v_{3}\right)}^{2}-3\left(v_{1}v_{2}+v_{1}v_{3}+v_{2}v_{3}\right)-\frac{3\beta }{k\gamma }<0.$$ (19)

Using bifurcation analysis (see S1 Appendix), we identify the following condition under which the FHN model has an unstable equilibrium and also a limit cycle:

$$\frac{v_{1}+v_{2}+v_{3}-\sqrt{\Delta_{2}}}{3}<v_{m}^{*}<\frac{v_{1}+v_{2}+v_{3}+\sqrt{\Delta_{2}}}{3},$$ (20)

where the parameters *v*_1_, *v*_2_, *v*_3_, *k*, *γ*, *ε*_1_, *ε*_2_, *c*_m_ should satisfy

$$\Delta_{2}={\left(v_{1}+v_{2}+v_{3}\right)}^{2}-3\left(v_{1}v_{2}+v_{1}v_{3}+v_{2}v_{3}\right)-\frac{3\varepsilon_{1}\varepsilon_{2}c_{m}\gamma }{k}>0.$$ (21)

Note that the parameters need to satisfy both Δ_1_ < 0 and Δ_2_ > 0, which introduces the following relationship:

$$\beta >\varepsilon_{1}\varepsilon_{2}c_{m}\gamma^{2}.$$ (22)

With Eq (18) and inequalities Eqs (19)–(21), we finally obtain the set of stimulus amplitude *ν* that leads to a limit cycle.

A periodic bursting-type action potential can then be derived by designing a stimulus amplitude so that the state of the FHN model periodically switches between a stable equilibrium and a limit cycle, hence producing a periodic spike trains. Here, we model the pacemaker activity ***J***_stim_ using a periodic function with stimulus amplitude *ν* in the limit cycle range (a value satisfying Eq (18) and inequality Eqs (19)–(21)):

$$J_{\text{stim}}=\frac{1}{\varepsilon_{1}}\nu h\left(t\right),$$ (23)

where *h*(*t*) = *h*(*t* + *T*), which is a periodic function with frequency *f* = 1/*T* that controls the time interval between trains of spikes and therefore contractions.

#### Frequency of bursting-type action potential

Having an analytical expression that relates the spike frequency of a bursting-type action potential with model parameters is useful to design realistic action potentials. The FHN system exhibits oscillatory solution when it has a limit cycle other than an unstable equilibrium. With the analysis of eigenvalues during bifurcation analysis (see S1 Appendix), the frequency of FHN model in a limit cycle is

$$\omega =\sqrt{\frac{\varepsilon_{2}}{\varepsilon_{1}c_{m}}}g\left(v_{1},v_{2},v_{3},k,\beta ,\delta ,\gamma ,\nu \right),$$ (24)

where *g*(*v*_1_, *v*_2_, *v*_3_, *k*, *β*, *δ*, *γ*, *ν*) is a function of *v*_1_, *v*_2_, *v*_3_, *k*, *β*, *δ*, *γ*, *ν*. The frequency is proportional to the square root of *ε*_2_ and inversely proportional to the square root of *ε*_1_. We can therefore enhance the frequency of spike trains simply by either decreasing *ε*_1_ or increasing *ε*_2_.

### Random conductivity tensor model

In this section, we introduce a random conductivity tensor model that aims at better capturing the lack of global structure of uterine fibers. At the tissue level, the velocity of the transmembrane potential propagation is dependent on the myocyte fiber anisotropy. In order to take into account the myometrial fiber orientation, we assume a regular fiber structure, that is, we define ***a***_*l*_(***r***) as a unit vector parallel to the main fiber orientation at a point ***r***. Following the same procedure as that in our previous work [14], the conductivity tensors in a global coordinate system are given by

$$G_{i}^{{}^{\prime }}\left(r\right)=\left(\sigma_{\text{il}}-\sigma_{\text{it}}\right)a_{3}\left(r\right)a_{3}^{T}\left(r\right)+\sigma_{\text{it}}I_{3},$$ (25)

$$G_{e}^{{}^{\prime }}\left(r\right)=\left(\sigma_{\text{el}}-\sigma_{\text{et}}\right)a_{3}\left(r\right)a_{3}^{T}\left(r\right)+\sigma_{\text{et}}I_{3},$$ (26)

where $G_{i}^{{}^{\prime }}\left(r\right)$ ($G_{e}^{{}^{\prime }}\left(r\right)$) is the intra (extra) cellular conductivity tensor, *σ*_il_, *σ*_it_ (*σ*_el_, *σ*_et_) are the longitudinal and transversal conductivity values of the intra (extra) cellular domain, respectively, ***a***_3_(***r***) is a basis vector that is parallel to ***a***_*l*_(***r***), and ***I***_3_ denotes an identity matrix of size 3 × 3.

**Fig 2:**
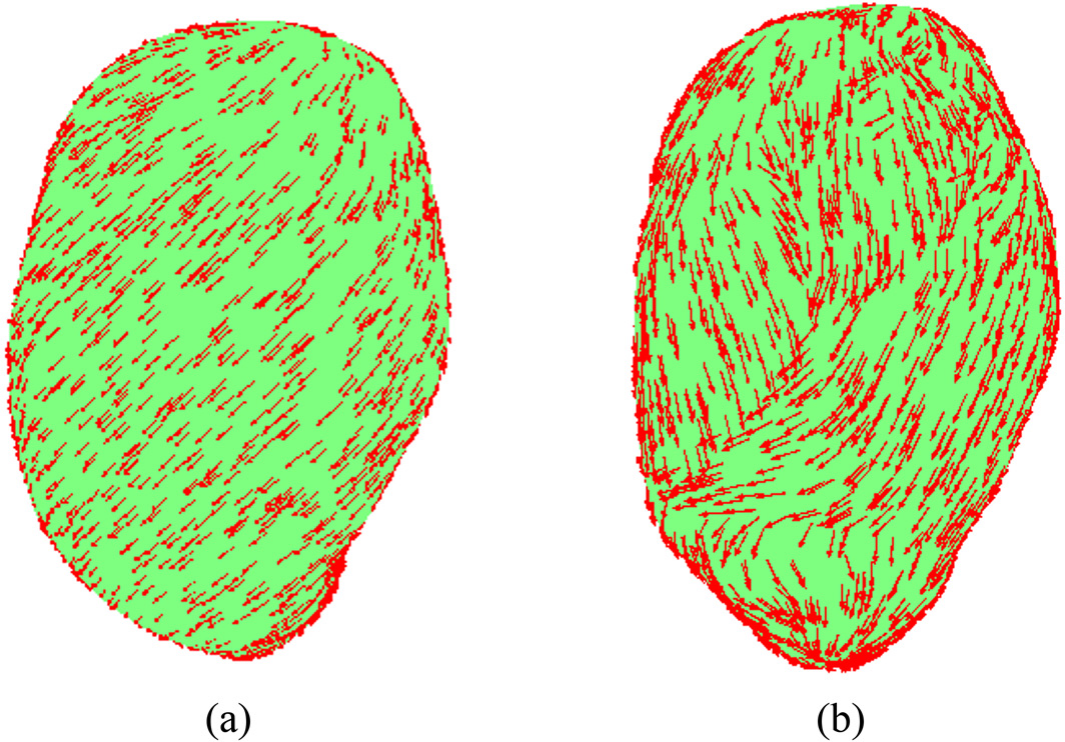
Anatomically accurate uterus model. (a) Uterus model with fixed conductivity tensor angle $\frac{\pi }{4}$. (b) Uterus model with randomly sampled conductivity tensor angles.

In our previous work [14], we represented the uterus as a hollow volume with uniform thickness and introduced a general framework for designing the tensor direction in the myometrium for an arbitrary uterine shape. Specifically, we presented the fiber orientation ***a***_3_(***r***) using its angle *α* with respect to the vector defined in the tangential plane at that point ***r***. The angle *α* can be modeled as a spatial basis function defined over the whole uterine domain. In the numerical example, however, we only considered a fixed fiber angle of *π*/4 for the whole myometrium (Fig 2(a)). In this work, we define the fiber orientation using a finite element method (FEM) mesh. We divide the uterus into 25 contiguous regions, via random sampling of the finite elements within the myometrium model, resampling any point that lies less than 4 cm from its nearest neighbor. We compute the vector based on the surface normals at our uterus model mesh points for an arbitrary uterine shape. Given a mesh point ***r***, with surface normal $\hat{n}\left(r\right)={\left[n_{x},n_{y},n_{z}\right]}^{{}^{\prime }}$, the fiber orientation ***a***_3_(***r***) is defined as

$$a_{3}\left(r\right)=\left(\begin{array}{c} \frac{n_{x}n_{y}\cos\left(\alpha \right)-n_{z}\sin\left(\alpha \right)}{\sqrt{n_{x}^{2}+n_{z}^{2}}}\\ \sqrt{n_{x}^{2}+n_{z}^{2}}\cos\left(\alpha \right)\\ \frac{n_{z}n_{y}\cos\left(\alpha \right)-n_{x}\sin\left(\alpha \right)}{\sqrt{n_{x}^{2}+n_{z}^{2}}} \end{array}\right),$$ (27)

where *α* is the angle of the fiber relative to the tangent vector. In this work, the vertical axis is the y axis rather than the z axis in the previous work [14]. In order to take into account the spatial variation of fiber orientations, we assign random fiber angles to each region of uterus by sampling from the normal distribution (0, *π*/4) (Fig 2(b)), where 0 is oriented along the vertical axis of the uterus.

### Speed of propagation for bursting-type potential

In our previous work [14], we obtained the expression for anisotropy ratio *ζ* by solving for a traveling wave solution with reported value of propagation speed of plateau-type potential. In this section, we derive the speed of propagation of the bursting-type potential by following a similar solution for the traveling wave with fixed value of *ζ*.

A traveling wave is defined as a wave which travels without change of shape. The speed of propagation of the leading front of the waveform can then be taken as the wavespeed. Considering the leading front of a transmembrane potential, the variable *w* of the FHN model changes slowly, which is then regarded to be set at the resting value. With Eq (17), we get its resting value as follows:

$$w=\frac{1}{\gamma }\left(\beta v_{\text{mr}}+\delta \right),$$ (28)

where *v*_mr_ is the resting potential. Replacing Eq (28) in Eq (6), the reaction diffusion equation is then presented by

$$\frac{\partial v_{m}}{\partial t}=\frac{k}{\varepsilon_{1}c_{m}}\left(\left(v_{m}-v_{1}\right)\left(v_{2}-v_{m}\right)\left(v_{m}-v_{3}\right)-\frac{1}{k\gamma }\left(\beta v_{\text{mr}}+\delta \right)\right)+\frac{\zeta }{\zeta +1}\frac{\sigma_{\text{el}}}{a_{m}c_{m}}\frac{\partial^{2}v_{m}}{\partial l^{2}},$$ (29)

where *a*_m_ is the surface-to-volume ratio of the membrane. Eq (29) matches the standard form of reaction diffusion equation for an excitable kinetics model in [22], whose wavespeed is given by

$$c=\frac{{\tilde{v}}_{1}-2{\tilde{v}}_{2}+{\tilde{v}}_{3}}{c_{m}}{\left(\frac{k\sigma_{\text{el}}\zeta }{2\varepsilon_{1}a_{m}\left(\zeta +1\right)}\right)}^{1/2},$$ (30)

where ${\tilde{v}}_{1}$, ${\tilde{v}}_{2}$, and ${\tilde{v}}_{3}$ are the roots of the following polynomial

$$f\left(v_{m}\right)=\left(v_{m}-v_{1}\right)\left(v_{2}-v_{m}\right)\left(v_{m}-v_{3}\right)-\frac{1}{k\gamma }\left(\beta v_{\text{mr}}+\delta \right).$$ (31)

Note that the speed of propagation is proportional to the square root of extracellular longitudinal conductivity *σ*_el_ and inversely proportional to membrane capacitance *c*_m_ and the square root of surface-to-volume ratio *a*_m_.

### Realistic volume conductor model

**Fig 3:**
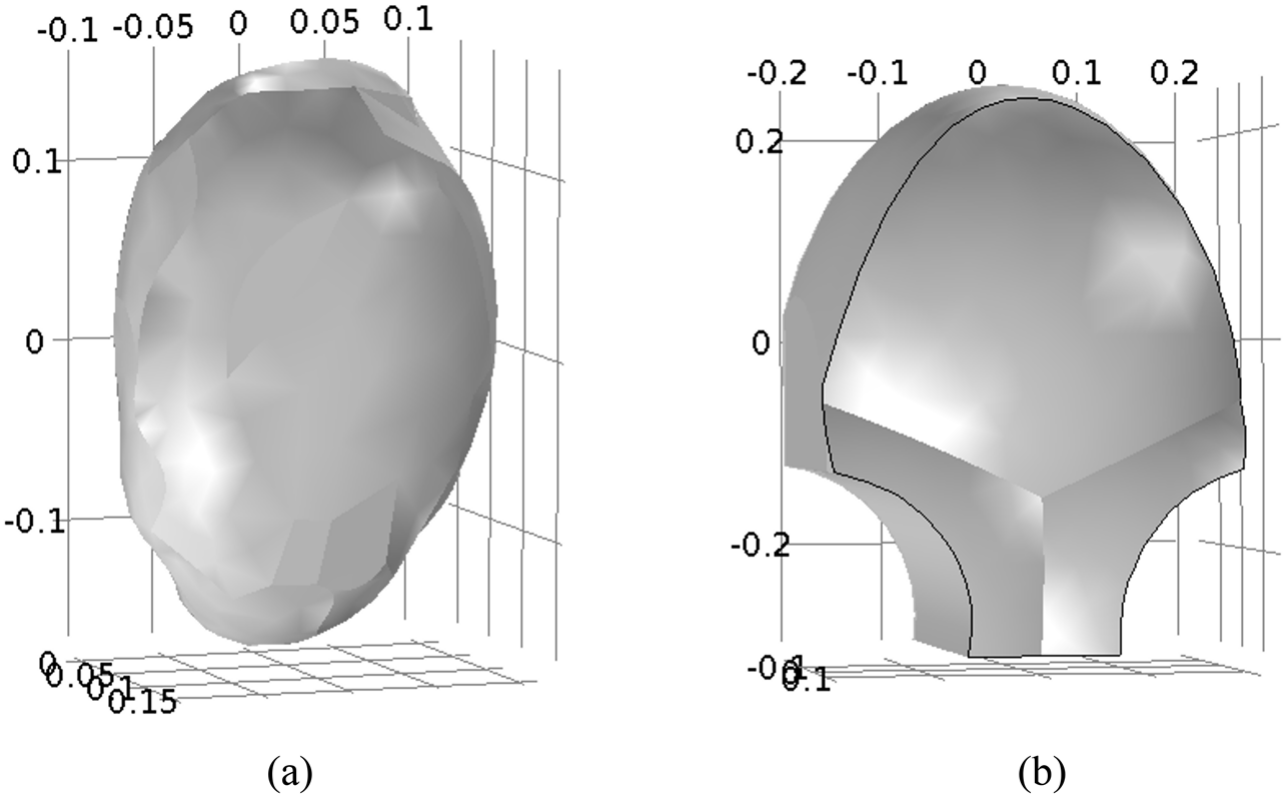
Realistic uterus and abdomen model. (a) Outer surface of anatomical uterus. (b) Outer surface of SARA-based abdomen model (in meters).

At the organ level, we create anatomically realistic models for the volume conductors. Rather than using a simple spherical model for the uterus, we model the uterus based on the MRI of a real, near-term, pregnant woman. We adapt a uterine mesh from the FEMONUM project (available through http://femonum.telecom-paristech.fr/downloadPage.html) [23], creating a smooth, 3D model for the organ with a uniform 1cm thickness (Fig 3(a)). Similarly, rather than using a spherical model for the abdomen, we assume that, when the mother leans against the device, her abdomen will deform slightly to follow the device contours. Following this assumption, we create an abdominal model that follows the shape of the SARA device, offset from the front of the uterus by roughly 2 cm (Fig 3(b)).

### Sensor model on the abdominal surface

Finally, in order to obtain a more accurate simulation of magnetic field measurements especially around the lower abdomen, we replicate the true sensor positions and measurements from the SARA device, rather than simply observing the normal component of the magnetic field at the abdomen. The SARA sensors are hardware gradiometers; each sensor measures the magnetic field at two coils. The first is on the inner surface of the device close to the mother’s abdomen (denoted as *B*_1_) and the second is displaced 8 cm from the first in a direction roughly normal to the SARA surface (denoted as *B*_2_). The recorded SARA measurement from the *i*th sensor is actually the difference between the fields at these two points, which is given by

$$B_{i}=B_{i1}-B_{i2},$$ (32)

where *i* = 1, 2, ⋯, 151. This recording is very similar, but not identical, to the normal component of the magnetic field. The discrepancy is particularly notable in areas such as the lower central portion of the SARA device where the convexity of the surface results, due to space constraints in placing the sensors, in a non-negligible difference between the surface normal and the actual sensor orientation.

### Summary of modeling assumptions

In this section, we list all the assumptions that are used to formulate the model:

1. Cellular level
  The uterus is formed by the same type of cells (myocyte) with cylindrically symmetric electrical conductivity. Any myocyte can act as pacemaker or pacefollower. The plateau-type and bursting-type action potentials are initiated by one pacemaker in the form of a stimulus current.
2. Tissue level
  Although there is no well-ordered global fiber structure, the fibers in localized regions of uterus have similar orientations. The conductivities of intracellular and extracellular domains are inhomogeneous and anisotropic with equal anisotropic ratio. The transmembrane potential travels across the myometrium without change of shape so that it can be defined as a traveling wave.
3. Organ level
  The volume conductor is modeled as four compartments with electrically conductive boundaries between compartments except for the external boundary of abdomen. When leaning against the SARA device, the abdomen will deform to follow the device shape. The anatomically realistic uterus with a layer of uniform-thickness wall is developed based on the MRI of a near-term, pregnant woman. The uterus has inhomogeneous and anisotropic conductivity while the other three compartments are considered to be homogeneous and isotropic.
4. Electromagnetics
  The electromagnetic property of uterine contraction is modeled using the quasi-static approximation of the Maxwell Equations [24, 25]. We can use this approximation because bioelectromagnetic fields vary slowly with frequency below 1 KHz and the corresponding spatial length scale is much larger than the volume conductor of geometry. Changes in the bioelectric sources, therefore, affect the bioelectromagnetic fields instantaneously in the whole volume conductor of geometry. The displacement current, which is the result of time-varying electric field, is much smaller than the ohmic current that results from ions flowing in the medium. Hence, the total current density in the volume conductor of geometry can be regarded as the ohmic current only.

### SARA data collection

The data presented here was collected at the University of Arkansas for Medical Sciences (UAMS). The protocol was approved by the UAMS Institutional Review Board. The study protocol was explained to the subjects and written consent to perform the study was obtained. The subjects were then requested to sit comfortably and lean forward on to the sensor array.

The duration of a recording was typically around 20 minutes with a sampling rate of 250 Hz. The MMG data was then downsampled to 32 Hz and processed using a bandpass filter of 0.1 − 1 Hz. Further, in order to exclude maternal breathing artifact, a notch filter of 0.25 − 0.35 Hz was applied. Using spectral analysis, the primary magnetic activity of a uterine contraction is represented by a low frequency band between 0.1 Hz and 0.4 Hz [26, 27]. The MMG activity in this range likely represents the plateau and repolarization phase of the action potentials. For a more accurate analysis that is closer to the real time-frame, a higher band 0.4 − 1 Hz should be added to the analysis [26, 27]. Also, early studies [28–30] have shown that the power spectrum density of uterine EMG bursts in patients during active labor is peaked at 0.71±0.05 Hz as compared to non-laboring term 0.48 ± 0.03 Hz patients. Most of the uterine EMG studies apply a bandwidth of 0.35 − 1 Hz. The lower band limit of 0.35 Hz is chosen to avoid movement artifacts and also since maternal breathing is a prominent signal around the frequency of 0.33 Hz which can contaminate uterine EMG acquisition. In our study, we included the frequency band 0.1 − 0.25 Hz since we have the ability to acquire signals as low as 0.1 Hz with a relatively high signal to noise ratio with the SARA system [15, 16].

## Results

In this section, we evaluate our multiscale forward model at the cellular, tissue, and organ levels.

### Cellular level

**Fig 4:**
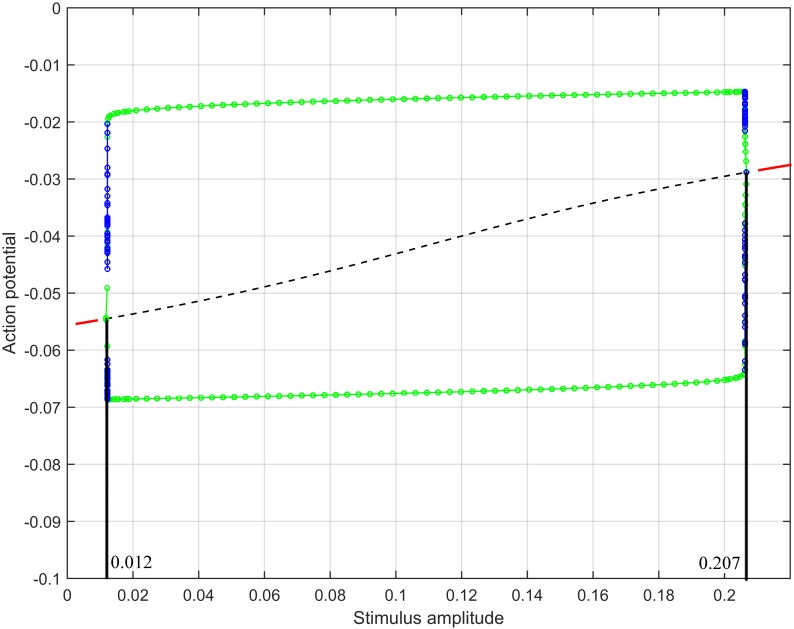
Bifurcation diagram, produced by XPPAUT, of action potential with the variation of stimulus amplitude.

The plateau-type action potential under certain parameters was illustrated in our previous paper [14]. To generate a bursting-type action potential, we computed the range of stimulus amplitude such that the FHN system, with model parameters as in Table 2, underwent a limit cycle behavior. Specifically, Δ_1_ in this case is negative and Δ_2_ is positive, hence by evaluating Eq (18) and inequality Eq (20), we obtained that *ν* should range between 0.012 and 0.207. We confirmed our computation with the bifurcation diagram (Fig 4) produced by XPPAUT [31] which is an effective numerical tool for simulating, animating, and analyzing dynamical systems. The bifurcation parameter *ν* is shown on the horizontal axis and the vertical axis shows the values of the function. The red lines denote stable equilibria while the black lines are the unstable equilibria. The green solid circles represent stable limit cycles while the blue ones refer to the unstable limit cycles. Note that limit cycle behavior is produced when the stimulus amplitude *ν* is between the two vertical lines.

**Table 2:** Ionic current model parameters.

Symbol	Value
*c*_m_	0.01[F/m^2^]
*ε*_1_	10[Ωm^2^]
*ε*_2_	10[1/S]
*v*_1_	-0.02[V]
*v*_2_	-0.04[V]
*v*_3_	-0.065[V]
*k*	7000[1/V^2^]
*δ*	0.052[V]
*γ*	0.1
*β*	1

**Fig 5:**
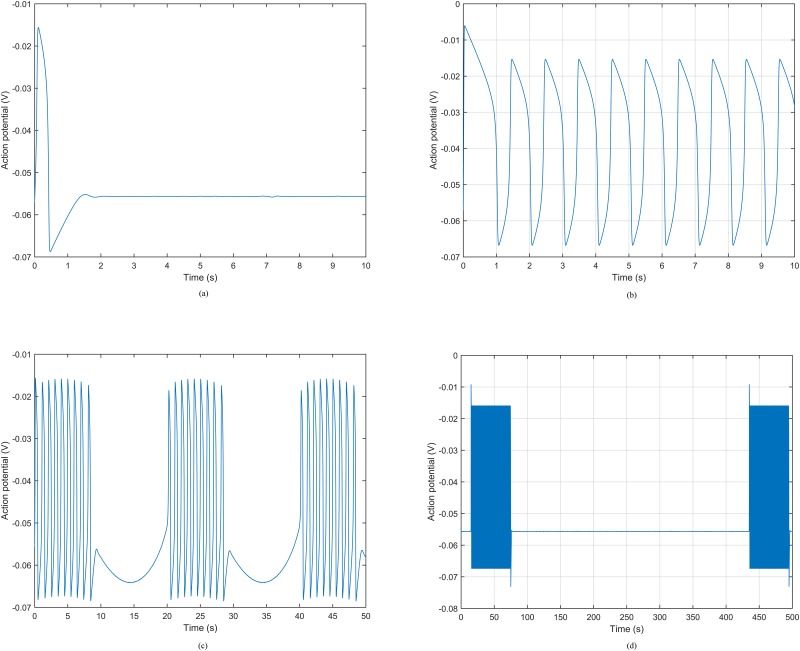
Action potentials by applying different stimuli. (a) Plateau-type action potential. (b) Periodic spike trains. (c) Periodic bursting-type action potential with a sinusoidal stimulus. (d) Periodic bursting-type action potential with a periodic heaviside-function stimulus.

The ionic current model is a nonlinear dynamical system of ordinary differential equations which should be solved numerically. We used MATLAB’s built-in ode45 function, which includes fourth order Runge-Kutta method, to validate the cellular level model. We reproduced different types of action potentials (Fig 5) by varying the stimulus current. Fig 5(a) shows the plateau-type action potential, which was obtained by applying no stimulus current. In this case, a stable equilibrium exists in the FHN model. The system returns to the equilibrium point after experiencing an excitation, generating the plateau-type action potential. If the stimulus current is set to be 0.15 A/m^2^, it falls in the limit cycle range and hence periodic spike trains will be generated (Fig 5(b)). Periodic stimulus current that changes between stable equilibrium and limit cycle ranges gives rise to the periodic bursting-type action potential. Fig 5(c) shows an example of the periodic bursting-type action potential by applying a sinusoidal stimulus current with amplitude *ν* = 0.11 and frequency *f* = 0.05 Hz in Eq (23).

We designed the stimulus current of the generalized FHN model using as a reference the average bursting-type action potential recorded from isolated tissue strips of human myometrium at term [20]. In particular, as reported in [20] from the 39th week of pregnancy, the resting potential is approximately −56 mV and the interval between contractions is around 7 minutes. In this numerical example, we introduced the heaviside function, which is the unit step function whose value is zero for negative argument and one for positive argument, as the stimulus current. With a periodic heaviside function whose duration is 1 minute and period is 7 minutes, the periodic bursting-type action potential is illustrated in Fig 5(d). We observe that the resting potential of our model is −55.6 mV, and the period of consecutive contractions is about 420 *s*, which are in fair agreement with the recorded values in [20].

In Table 3, we examined the relationship between the frequency of spike trains and parameters *ε*_1_ and *ε*_2_ using the stimulus current in Fig 5(b). We generated transmembrane potentials for different combinations of *ε*_1_ and *ε*_2_ values. It shows that we are able to obtain a higher frequency with smaller *ε*_1_ and larger *ε*_2_, which is consistent with the relationship that is derived in Eq (24).

**Table 3:** Frequency of spike trains (spiking/second).

***ε*_1_**	**1**	**2**	**3**	**4**	**5**	**6**	**7**	**8**	**9**	**10**
***ε*_2_**										
**1**	0.164	0.161	0.159	0.156	0.154	0.151	0.149	0.147	0.145	0.143
**2**	0.333	0.323	0.313	0.302	0.294	0.286	0.278	0.270	0.263	0.256
**3**	0.488	0.458	0.444	0.431	0.429	0.425	0.408	0.400	0.392	0.377
**4**	0.606	0.588	0.571	0.556	0.541	0.532	0.526	0.510	0.502	0.495
**5**	0.769	0.741	0.714	0.690	0.667	0.645	0.625	0.606	0.600	0.581
**6**	0.870	0.847	0.833	0.800	0.769	0.741	0.733	0.720	0.707	0.693
**7**	1.042	1.000	0.947	0.918	0.882	0.861	0.840	0.833	0.800	0.769
**8**	1.200	1.190	1.087	1.020	1.000	0.962	0.926	0.893	0.861	0.820
**9**	1.333	1.277	1.200	1.136	1.111	1.064	1.020	1.000	0.980	0.943
**10**	1.556	1.429	1.304	1.250	1.200	1.154	1.111	1.071	1.034	1.020

### Tissue level

Table 4 gives the parameter values (other than those included in Table 2) for the analysis of propagation speed of the bursting-type potential. According to Eq (30), the speed of propagation is 0.0429 m/s along the main fiber direction. In order to verify the speed, the fiber orientation *α* in Eq (27) was set to be 0 so that the fibers lay along the vertical axis of the uterus. In our numerical simulation, we took the difference between the top and equator of uterus and find the speed at the equator is approximately 0.0421 m/s, which is a good match with the above theoretic value of wavespeed.

**Table 4:** Parameters for speed analysis.

Symbol	Value
*a*_m_	575870[m^-1^]
*v*_mr_	-0.056[V]
*σ*_el_	0.68[S/m]
*ζ*	0.518

### Organ level

**Fig 6:**
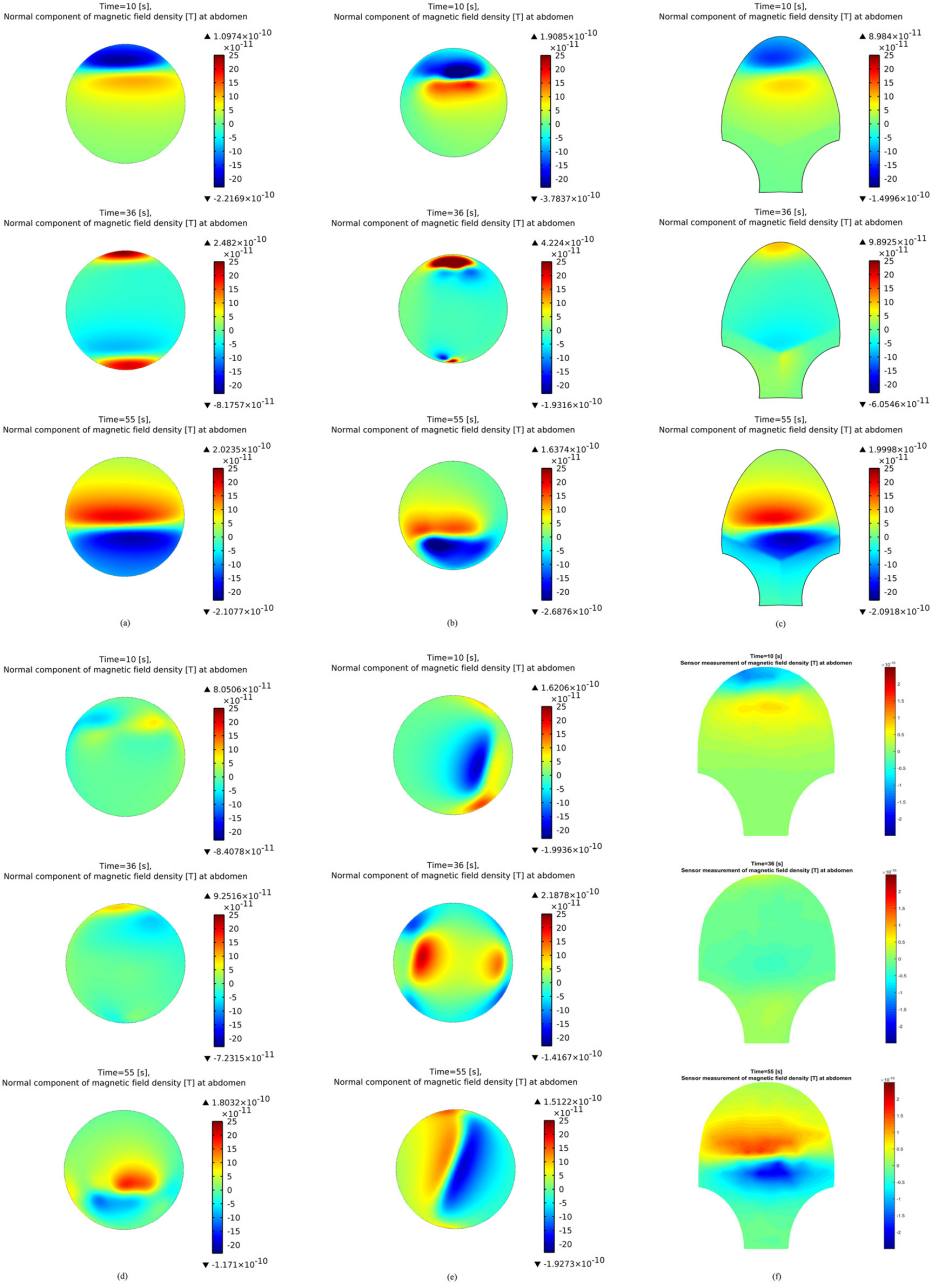
Magnetic field simulations at the abdominal surface at time instants *t* = 10 s, 36 s, 55 s. (a) Simulated normal magnetic field with original configuration. (b) Simulated normal magnetic field with anatomical uterus. (c) Simulated normal magnetic field with SARA-shape abdomen. (d) Simulated normal magnetic field with random fiber orientation. (e) Simulated normal magnetic field with pacemaker set at the lateral of uterus. (f) Simulated magnetic field with SARA-shape abdomen and sensor model.

We tested various configuration aspects, e.g., shape of uterus, shape of abdomen, fiber orientation, and pacemaker location, by simulating the abdomen-surface magnetic field using our multiscale forward model. Fig 6 presents simulations of the abdominal magnetic field under each configuration (the detailed configuration for each subfigure is shown in Table 5). In [14], we illustrated the normal magnetic field simulation for one pacemaker on the fundus of a spherical myometrium with fixed-angle fiber and a spherical abdomen (Fig 6(a)). In order to investigate the sensitivity of MMG to each aspect in configuration, we simulated the normal magnetic field with one aspect of configuration changed in each numerical example (Fig 6(b)–6(e)) when compared with the original normal magnetic field (Fig 6(a)) that was obtained using the simplified model. Fig 6(b) was generated by introducing an anatomical uterus developed from the FEMONUM project instead of a spherical uterus. It can be seen that the general pattern of normal magnetic field for the anatomical uterus is similar to that for a spherical one. The normal magnetic field pattern over a SARA-shape abdomen is shown in Fig 6(c). We observe that no significant difference is introduced into the abdominal magnetic field even though the shape of abdomen is changed. Fig 6(d) illustrates the normal magnetic field with fiber orientation randomly taken from a normal distribution, which displays a significantly different pattern from that with fixed-angle fiber orientation. When the pacemaker is located at the lateral of uterus, the normal magnetic field pattern is given in Fig 6(e). It is obvious that the propagation direction of magnetic field is influenced by the location of pacemaker. Fig 6(f) presents the magnetic field pattern applying the sensor model, which replicates the true SARA sensor readings, to the same configuration as in Fig 6(c). It is shown that significant difference of magnetic field pattern occurs at the lower abdomen.

**Table 5:** Detailed model configurations for Fig 6.

Configuration	Shape of uterus	Shape of abdomen	Fiber orientation	Pacemaker location	Sensor model
No.					
**a**	Sphere	Sphere	Fixed-angle	Fundus	No
**b**	FEMONUM	Sphere	Fixed-angle	Fundus	No
**c**	Sphere	SARA-shape	Fixed-angle	Fundus	No
**d**	Sphere	Sphere	Random	Fundus	No
**e**	Sphere	Sphere	Fixed-angle	Lateral	No
**f**	Sphere	SARA-shape	Fixed-angle	Fundus	Yes

### Initial validation with real data

Matching a simulation of a full contraction to a real contraction would require optimization of many variables, such as the initial pacemaker location, pacemaker intensity, and fiber directions, and taking into consideration complex scenarios such as one transmembrane potential taking a tortured path that travels across the whole uterus [32], or a transmembrane potential that travels confined within a restricted area and recruits other pacemakers in areas across the uterus [33, 34]. In the second scenario, the locations and activation times of the recruited pacemakers must also be optimized. Without an inverse model, manually optimizing these variables is infeasible. As such, we validate our model by comparing its magnetic field outputs with the real patient MMG data during a limited portion of a uterine contraction. Specifically, we match the emergence and decay of the contraction, when it is known that the contraction will be limited to a small region around a single pacemaker.

**Fig 7:**
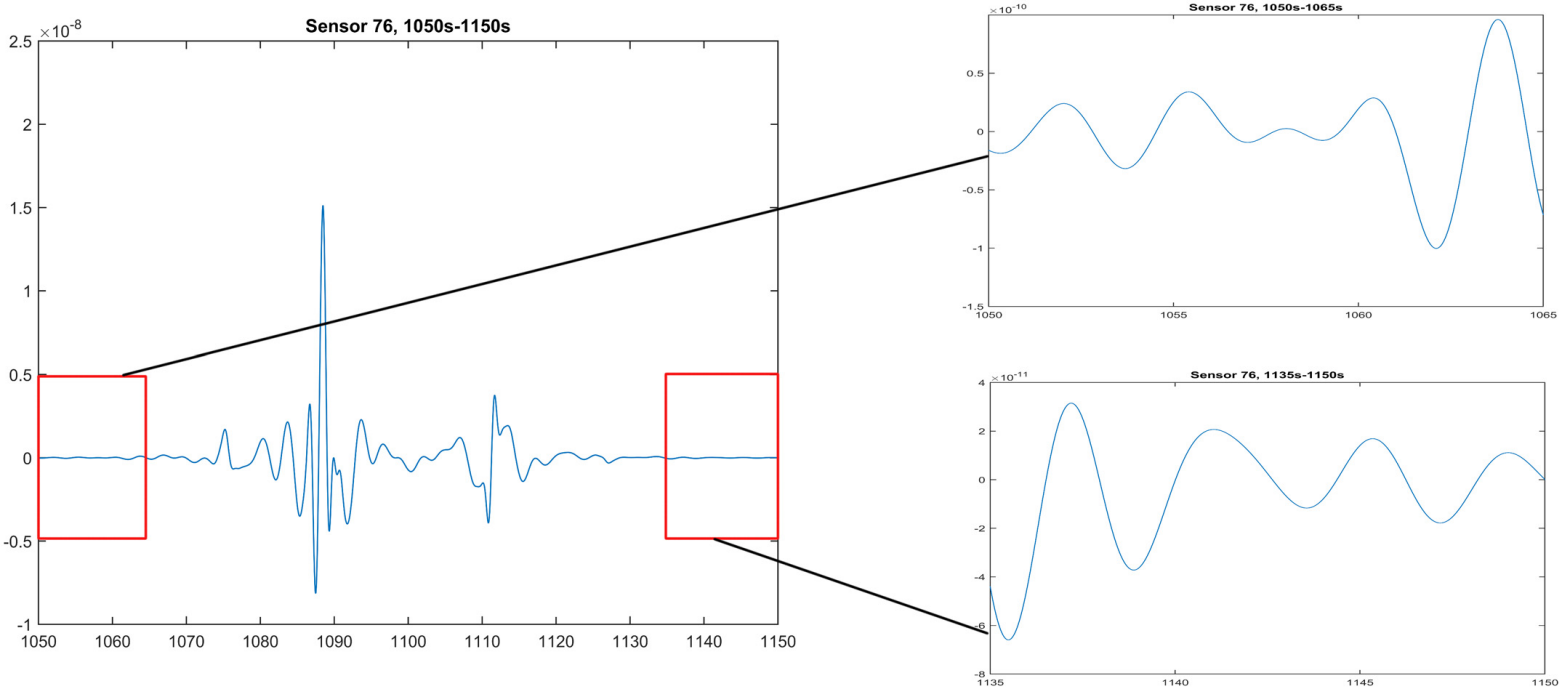
The emergence and decay portions (1050 s–1065 s, 1135 s–1150 s) of real patient data collected by sensor 76, which is at the top of SARA device.

The real data presented here was collected from a pregnant woman whose gestational age was 38 weeks and 4 days (see SARA data collection for details). We chose to simulate the emergence and decay of one pre-labor uterine contraction, which are marked in the red boxes (1050 s–1065 s, 1135 s–1150 s) in Fig 7. Considering the alternating positive and negative magnetic field measurements in Fig 7, we decided to adopt the ionic current model that can generate the bursting-type action potential.

In our numerical example, we applied the simplified spherical uterus with a 16 cm radius measured from the center to the external boundary surface of the myometrium ∂M and assumed the uterine wall with a uniform thickness of 1 cm. The spherical abdominal compartment with a 21 cm radius was 2 cm offset from the front of the uterus. The spherical uterus and abdomen were chosen according to the above analysis at the organ level that the geometry has little impact in the magnetic field pattern when compared to those of the real anatomy and SARA shape. We also defined the fetus to be a solid sphere with a 12 cm radius concentric to the myometrium. The conductivity values of the homogeneous and isotropic compartments were listed in Table 6. The anisotropy of uterus was represented by the random conductivity tensor model and the surface normals were specified relative to the mesh points of the uterus. We randomly chose the conductivities of uterus to be either 0 or sampled from a normal distribution such that the average value matched the conductivity value in Tabel 4. We assumed that one pacemaker was located at the back of the uterus, centered at (0, 13.0, −7.5)cm, which was represented by $J_{\text{stim}}\left(r,t\right)=\frac{1}{\varepsilon_{1}}*V_{\text{stim}}*\text{heaviside}\left(t\right)*\text{heaviside}\left(T_{\text{stim}}-t\right)*\text{heaviside}\left(0.05-{\left({\left(y-\cos\left(-\frac{\pi }{6}\right)*0.15\right)}^{2}+{\left(z-\sin\left(-\frac{\pi }{6}\right)*0.15\right)}^{2}+x^{2}\right)}^{1/2}\right)*\cos\left(2\pi *0.05*\left(t-T_{\text{stim}}\right)\right)$ with stimulus amplitude *V*_stim_ = 0.11 V and stimulus duration *T*_stim_ = 5 s.

**Table 6:** Conductivity values of the homogeneous and isotropic compartments.

Symbol	Value
$G_{A}$	0.2[S/*m*]
$G_{U}$	1.74[S/*m*]
$G_{F}$	0.5[S/m]
*G*_air_	5*10^−15^[S/m]
*ζ*	0.818

The limited propagation of the bursting-type transmembrane potential, as expected during the emergence and decay of a new contraction, can be achieved either by simply electrically isolating the limited propagation area or, more realistically, by having region-specific ionic current model parameters. To test the more realistic limited-propagation approach, we set the ionic current model parameters to lie within the limit cycle range for the region of uterus less than 10 cm from the center of the pacemaker, and designed the parameters within the stable equilibrium range outside this region of uterus (see Table 7 for detailed parameters).

**Table 7:** Region-specific ionic current model parameters.

Symbol	Value within propagation area	Value outside propagation area
*c*_m_	0.01[F/m^2^]	0.01[F/m^2^]
*ε*_1_	10[Ωm^2^]	200[Ωm^2^]
*ε*_2_	10[1/S]	0.09[1/S]
*v*_1_	-0.02[V]	-0.02[V]
*v*_2_	-0.04[V]	-0.04[V]
*v*_3_	-0.065[V]	-0.065[V]
*k*	7000[1/V^2^]	10000[1/V^2^]
*δ*	0.052[V]	0.052[V]
*γ*	0.1	0.1
*β*	1	1

The computation of the electromagnetic fields on the uterine and abdominal surfaces was implemented using the FEM solver COMSOL Multiphysics version 4.3a on a server with 12 processors at 2.3 GHz with 64 GB RAM. We discretized the four-compartment volume conductor geometry into 1,181,186 tetrahedral elements, which were allocated as: 109,237 elements in the additional compartment concentric to the abdomen with a 50 cm radius, 321,633 elements in the abdominal cavity, 555,180 elements in the myometrium, 190,289 elements in the intrauterine cavity, and 4,847 elements in the fetus. The uterine and abdominal surfaces were divided into 38,844 and 6,834 triangular elements, respectively. After the discretization, the computations of transmembrane potential, electrical potential, and magnetic field were given in 3 steps. The first step to solve the 10-minutes uterine transmembrane potential required 4.5 hours when the number of degree of freedom was 223,274, while the solution time in the second step for electrical potential was 7 hours with number of degree of freedom 1,440,961. In the computation of abdominal magnetic field, the number of degree of freedom was 1,579,337 and the corresponding solution time was 4 hours when we chose the generalized minimal residual (GMRES) solver.

**Fig 8:**
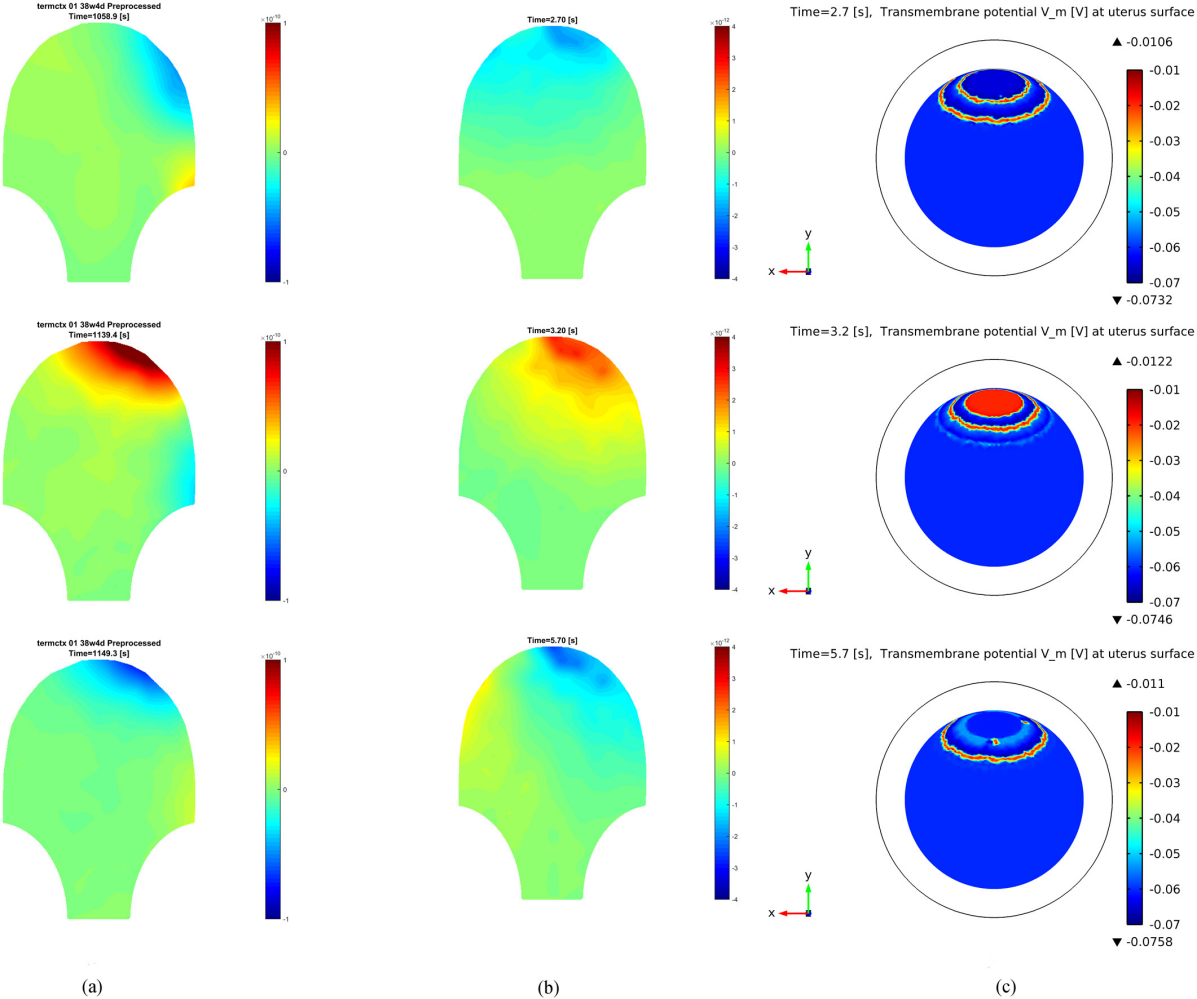
Real patient MMG data and FEM solution of our forward model at different time instants. (a) Real patient data. (b) Simulated magnetic field at the abdominal surface. (c) Transmembrane potential at the uterus surface.

Fig 8 shows several snapshots of the real patient MMG and the FEM solution of our forward model at three time instants within the limited propagation mode of a contraction (i.e., during the beginning or end of a contraction). Fig 8(a) provides the magnetic field measurements of the pregnant woman during the two portions of the uterine contraction. Fig 8(b) illustrates the simulated magnetic field at the abdominal surface and the corresponding transmembrane potential at the uterus surface is shown in Fig 8(c). We observe that our multiscale forward model is able to capture certain features of the specific patient’s MMG recordings, such as the spreading pattern of magnetic field at different areas, by proper placement of the pacemaker and restriction of the propagation area.

## Discussion

As an ionic current model, the FHN equations produced a fair approximation of the plateau-type action potential [14]. We generated a periodic bursting-type action potential by applying a periodic stimulus current into the FHN equations. Our simulation result at the cellular level shows that the simple spikes in a burst depolarize to an average of −25 mV. However, the depolarization of bursting-type action potential in human myocytes reported in [20] is much smaller (−42 mV). Note that in [14], the authors pointed out that the model parameters *v*_1_, *v*_2_, *v*_3_, and *k* control the range of *v*_m_, which means that it is possible to generate a bursting-type action potential as in the literature through adjusting these four parameters.

In our previous work, the magnetic field was recognized as highly dependent on the fiber orientation of the myometrium. Our numerical simulation results (Fig 6(a) and 6(d)) provide a fair illustration that the direction and intensity of magnetic field change significantly under different fiber orientations. The magnetic field recordings of pregnant women obtained using the SARA device also demonstrate the relationship between the pattern of the magnetic field and complex fiber orientations. In order to obtain the best match to patient recordings, fiber orientations should ideally be accurate to the individual. Unfortunately, a widely accepted model of fiber architecture of the myometrium is currently unavailable [35]. The current technique is to study the global fiber based on magnetic resonance (MR) diffusion tensor imaging (DTI). However, it is difficult to get approval for MRI studies on pregnant women that are not medically indicated, thus making it challenging to obtain an accurate fiber structure for every pregnant woman. Instead of totally random fiber orientation in this work, we can instead apply the average fiber structure of human myometrium, which is obtained from the MRDTI studies on the recruited subjects in this project.

In our work, we are assuming that the abdomen follows the contour of the SARA device after the pregnant woman sits and leans her abdomen against the device. This abdominal model would be incorporated into our entire modeling if it produces magnetic field that matches more with the real data. However, the magnetic field measured at the realistic abdomen (Fig 6(c)) shows minimal changes when compared with that measured at the spherical abdomen (Fig 6(a)). In this sense, a spherical abdomen is suitable to represent the geometry of the abdomen surface.

The spherical volume conductor geometry was thought to be an oversimplification of the real uterus and a more realistic model was proposed [14]. However, the simulation result with an anatomical uterus from the FEMONUM project (Fig 6(b)) displays a quite similar magnetic field pattern to that of the spherical one (Fig 6(a)). The spherical uterus assumption is appropriate for SARA device analysis, since the shape of the SARA device is concave, which is related to the shape of the abdomen. Despite specific uterine shapes at different stages of pregnancy, a spherical geometry is therefore a good choice for simplified modeling without causing too much distortion.

It is expected that each uterine myocyte can act as a pacemaker as well as a pacefollower. In this work, we observe that the location of pacemaker has significant impact on the magnetic field pattern (Fig 6(e)). The sensitivity of propagating direction to pacemaker locations makes it possible for us to determine from real data where the pacemaker is located. Exploring the configurations that characterize specific contraction patterns is exactly what we want to achieve by developing the multiscale forward model of contractions in pregnant uterus.

Obtaining a better replication of real data in a relatively simple way is possible with the following steps. Regarding the volume conductor geometry, we propose to use the simple spherical model no matter which stage the pregnant woman is in and how her abdomen is deformed. Accurate modeling for the conductivity tensor and pacemaker location is important since they have a more significant effect on the pattern of magnetic field. The fiber orientation should be averaged to population according to the MR DTI while the pacemaker can be set at the back of uterus. Periodic bursting-type action potential is introduced into our model by adding a periodic stimulus current in the FHN model. In [10], the ionic current model with a simple constant stimulus current produces more complex action potentials other than the bursting-type one simply by adjusting the parameters of the model, which makes it a perfect applicant as our ionic current model.

The application of our multiscale forward model to real MMG data shows that our model is able to mimic the limited-propagation magnetic field patterns during the emergence and decay of a uterine contraction. Note that this result is obtained by applying a pacemaker at the back of the uterus, a random fiber direction, a simplified spherical volume conductor geometry, and region-specific ionic current model parameters. Our model, therefore, is flexible to mimic the magnetic field patterns at different stages of uterine contraction by simply changing the aspects of model configuration. Among other things, our modeling approach enables us to test for different scenarios hypothesized in the literature regarding the recruitment of the uterus during a contraction [32–34].

Our first analysis on real data shows that reproducing the magnetic field patterns for the entire uterine contraction process would require estimating the model parameters for the specific subject and setting the right model configurations as described in Table 5. In fact, based on our results, the magnetic field patterns are sensitive to ionic current model parameters, conductivity tensor model of myometrium, and pacemaker location. To estimate the configurations that best predict the data of a particular patient, there is a need to formally solve the inverse problem of uterine contractions using the proposed multiscale model. Given the MMG measurements and our multiscale forward model, we can estimate the current density, conductivity tensor, and pacemaker location by solving the inverse problem. Developing the multiscale forward model is the prerequisite of solving the inverse problem while solving the inverse problem functions as the bridge between the multiscale modeling and clinical application.

Although solving the inverse problem is out of the scope of this paper, we are currently working on developing the statistical framework to enable this approach. We intend to first estimate the current density in the myometrium from the abdominal MMG measurements, with which we intend to discover the features that can characterize the preterm uterine contraction patterns. The performance of our model will be further quantified by root-mean-square error (RMSE) between the simulated magnetic field, which is obtained using our model with configuration properly set, and the real patient recordings.

## Conclusions

We developed a realistic multiscale forward model of contractions in pregnant uterus jointly at the cellular, tissue, and organ levels. Our approach incorporated the electrophysiological and anatomical knowledge of uterine contractions to compute the abdominal magnetic field. At the cellular level, we introduced a variation of the FitzHugh-Nagumo equations to generate both plateau-type and bursting-type action potentials. Analytical expressions for the speed of propagation and frequency of the bursting potential were derived and validated using numerical examples. We also designed a random conductivity tensor model applicable to an arbitrary uterine shape at the tissue level. At the organ level, we introduced a realistic anatomical model for the volume conductor geometry based on the magnetic resonance images of a pregnant woman. In order to simulate the real measurements of the SARA device, we proposed an array sensor model on the abdominal surface. Finally, we investigated the sensitivity of magnetic field pattern to the configuration aspects using numerical examples. Since the volume conductor geometry rarely changes the pattern of magnetic field, we conclude that the spherical shape stands out as a good approximation of the geometry of uterus and abdomen. We demonstrate that fiber orientation and pacemaker location have a great effect on the pattern of magnetic field. We also show that it is flexible to mimic the limited-propagation magnetic field pattern during the emergence and decay of the uterine contraction by setting certain configurations for our multiscale forward model.

For the future work, we will build up a conductivity tensor model based on magnetic resonance diffusion tensor imaging rather than wholly random as a better way to configure the fiber structure of uterus. We will also introduce a more complex ionic current model which considers the electrochemical characteristic of individual myocyte as in [10] and reproduce the full contraction simulation to match with the real patient data by estimating the optimal configurations using an inverse problem approach.

## Supporting Information

S1 Appendix Bifurcation analysis on a variation of the FHN model. (PDF)
