# Supplementary material for: Modeling Magnetomyograms of Uterine Contractions during Pregnancy Using a Multiscale Forward Electromagnetic Approach
Source: PLoS One. 2016 Mar 28;11(3):e0152421. doi: 10.1371/journal.pone.0152421 (PMC4809542; doi:10.1371/journal.pone.0152421)
Supplement: S1 Appendix — (PDF) [file pone.0152421.s001.pdf]

Bifurcation Analysis on a Variation of the FitzHugh-Nagumo (FHN) Model

Mengxue Zhang, Vanessa Tidwell, Patricio S. La Rosa, James D Wilson, Hari Eswaran, Arye Nehorai

Bifurcation analysis

The variation of the FHN equations is represented as

$$\frac{\partial v_{\mathrm{m}}}{\partial t} = \frac{1}{\varepsilon_1 c_{\mathrm{m}}}(k(v_{\mathrm{m}} - v_1)(v_2 - v_{\mathrm{m}})(v_{\mathrm{m}} - v_3) - w + \nu) \triangleq f_1(v_{\mathrm{m}}, w), \tag{1}$$

$$\frac{\partial w}{\partial t} = \varepsilon_2(\beta v_{\mathrm{m}} - \gamma w + \delta) \triangleq f_2(v_{\mathrm{m}}, w). \tag{2}$$

The nullclines of the FHN model are given by the following two equations

$$f_1(v_{\mathrm{m}}, w) = 0, \tag{3}$$

$$f_2(v_{\mathrm{m}}, w) = 0. \tag{4}$$

The v_{m}-nullcline is a cubic function of v_{m}, which is shown as

$$w = k(v_{\mathrm{m}} - v_1)(v_2 - v_{\mathrm{m}})(v_{\mathrm{m}} - v_3) + \nu, \tag{5}$$

and the w-nullcline is a straight line as follows:

$$w = \frac{\beta}{\gamma} v_{\mathrm{m}} + \frac{\delta}{\gamma}. \tag{6}$$

The equilibrium of the FHN system is the intersection of these two nullclines, which is defined as (v_{m}^*, w^*). The stimulus amplitude ν can then be denoted as a function of the

equilibrium $v_{\rm m}^*$ with other parameters fixed:

$$\nu(v_{\rm m}^*) = -k(v_{\rm m}^* - v_1)(v_2 - v_{\rm m}^*)(v_{\rm m}^* - v_3) + (\beta v_{\rm m}^* + \delta)/\gamma. \tag{7}$$

We are interested in the case when there is always only one equilibrium in the FHN system whatever the stimulus amplitude ν, for which the slope of the w-nullcline should be greater than the maximal slope of the $v_{\rm m}$-nullcline. The slope of the straight w-nullcline is $\dfrac{\beta}{\gamma}$, while the maximal slope of the $v_{\rm m}$-nullcline is found at the point $v_{\rm m} = \dfrac{v_1 + v_2 + v_3}{3}$ and it is equal to $\dfrac{k(v_1 + v_2 + v_3)^2}{3} - k(v_1 v_2 + v_1 v_3 + v_2 v_3)$. The condition for the existence and uniqueness of equilibrium is therefore given by:

$$\Delta_1 \triangleq (v_1 + v_2 + v_3)^2 - 3(v_1 v_2 + v_1 v_3 + v_2 v_3) - \frac{3\beta}{k\gamma} < 0. \tag{8}$$

The local stability of the equilibrium is determined by linearization of the nonlinear FHN model at the equilibrium, which is given by

$$\frac{\partial}{\partial t}\boldsymbol{x} = \boldsymbol{D}\boldsymbol{x}, \tag{9}$$

where $\boldsymbol{x} = \begin{pmatrix} v_{\rm m} - v_{\rm m}^* \\ w - w^* \end{pmatrix}$, and $\boldsymbol{D}$ is the Jacobian matrix at the equilibrium $(v_{\rm m}^*, w^*)$, which is shown as follows:

$$\begin{aligned} \boldsymbol{D} &= \left.\begin{pmatrix} \dfrac{\partial f_1(v_{\rm m}, w)}{\partial v_{\rm m}} & \dfrac{\partial f_1(v_{\rm m}, w)}{\partial w} \\ \dfrac{\partial f_2(v_{\rm m}, w)}{\partial v_{\rm m}} & \dfrac{\partial f_2(v_{\rm m}, w)}{\partial w} \end{pmatrix}\right|_{(v_{\rm m}^*, w^*)} \triangleq \begin{pmatrix} f_{1v_{\rm m}} & f_{1w} \\ f_{2v_{\rm m}} & f_{2w} \end{pmatrix} \\ &= \begin{pmatrix} \dfrac{q(v_{\rm m}^*, v_1, v_2, v_3, k)}{\varepsilon_1 c_{\rm m}} & -\dfrac{1}{\varepsilon_1 c_{\rm m}} \\ \varepsilon_2 \beta & -\varepsilon_2 \gamma \end{pmatrix}, \end{aligned} \tag{10}$$

where $q(v_{\rm m}^*, v_1, v_2, v_3, k) = -3kv_{\rm m}^{*2} + 2k(v_1 + v_2 + v_3)v_{\rm m}^* - k(v_1 v_2 + v_1 v_3 + v_2 v_3)$. The solution of Eq. (9) can be represented as

$$\boldsymbol{x}(t) = \exp(\lambda t)\ \boldsymbol{v}, \tag{11}$$

where λ is the eigenvalue of the Jacobian matrix $\boldsymbol{D}$ and $\boldsymbol{v}$ is its corresponding eigenvector. By solving the characteristic equation

$$\det(\boldsymbol{D} - \lambda \boldsymbol{I}) = 0, \tag{12}$$

we obtain the relationship between eigenvalue λ and the equilibrium v_{m}^{*}, which is given as follows:

$$\lambda^2 - (f_{1v_{\mathrm{m}}} + f_{2w})\lambda + (f_{1v_{\mathrm{m}}} f_{2w} - f_{1w} f_{2v_{\mathrm{m}}}) = 0. \tag{13}$$

Depending on the values of eigenvalues, we can determine whether the equilibrium is stable or not. Since we are interested in characterizing the stability of equilibrium as a function of model parameters, we need to compute eigenvalues as a function of these parameters and so we assess stability through the evaluation of two conditions as presented in Table 1.

Table 1. Conditions for the stability of equilibrium

**Type**	**Stability**	**Eigenvalues (λ_1 and λ_2)**	**Condition 1**	**Condition 2**
saddle	unstable	$\lambda_1 > 0,\ \lambda_2 < 0$ or $\lambda_1 < 0,\ \lambda_2 > 0$	$\lambda_1\lambda_2 < 0$	
node/focus	stable	$Re(\lambda_1) < 0,\ Re(\lambda_2) < 0$	$\lambda_1\lambda_2 > 0$	$\lambda_1 + \lambda_2 < 0$
node/focus	unstable	$Re(\lambda_1) > 0,\ Re(\lambda_2) > 0$	$\lambda_1\lambda_2 > 0$	$\lambda_1 + \lambda_2 > 0$

When the node or focus loses stability, it is possible to construct a bounding surface around the unstable equilibrium. According to the Poincaré-Bendixson theorem [1], a limit cycle must exit in the FHN system when the node or focus is unstable. In other words, the behavior of the FHN system changes qualitatively from a stable equilibrium to a limit cycle. Therefore, in order to identify the ranges of v_{m}^{*} so that the FHN system has a limit cycle, we need to compute condition 1 and condition 2 in Table 1 under which the node or focus is unstable, that is,

$$\begin{aligned} \lambda_1\lambda_2 &= f_{1v_{\mathrm{m}}} f_{2w} - f_{1w} f_{2v_{\mathrm{m}}} \\ &= -\varepsilon_2\gamma \frac{q(v_{\mathrm{m}}^{*}, v_1, v_2, v_3, k)}{\varepsilon_1 c_{\mathrm{m}}} + \frac{\varepsilon_2\beta}{\varepsilon_1 c_{\mathrm{m}}} > 0, \end{aligned} \tag{14}$$

$$\begin{aligned} \lambda_1 + \lambda_2 &= f_{1v_{\mathrm{m}}} + f_{2w} \\ &= \frac{q(v_{\mathrm{m}}^{*}, v_1, v_2, v_3, k)}{\varepsilon_1 c_{\mathrm{m}}} - \varepsilon_2\gamma > 0. \end{aligned} \tag{15}$$

With the condition (8), the discriminant of inequality (14) is negative. Therefore,

inequality (14) is satisfied for all v_{m}^* so that we only need to consider inequality (15). If the discriminant of the quadratic polynomial in inequality (15) is negative, the inequality is also satisfied for all v_{m}^*. In this work, we are interested in the case when the quadratic polynomial has positive discriminant, i.e.,

$$\Delta_2 > 0, \tag{16}$$

where $\Delta_2 = (v_1 + v_2 + v_3)^2 - 3(v_1 v_2 + v_1 v_3 + v_2 v_3) - \frac{3\varepsilon_1 \varepsilon_2 c_{\mathrm{m}} \gamma}{k}$, in which case inequality (15) is satisfied if

$$\frac{v_1 + v_2 + v_3 - \sqrt{\Delta_2}}{3} < v_{\mathrm{m}}^* < \frac{v_1 + v_2 + v_3 + \sqrt{\Delta_2}}{3}. \tag{17}$$

In a word, the equilibrium is unstable and instead the FHN system has a limit cycle if the equilibrium satisfies inequality (17) with the parameters v_1, v_2, v_3, k, β, γ, ε_1, ε_2, c_{m} satisfying inequalities (8) and (16). Together with Eq. (7) and inequality (17), we finally obtain the range of the stimulus amplitude ν to produce a limit cycle.

At the transition point (the point at which equilibrium loses stability), the real parts of eigenvalues vanish and the eigenvalues are

$$\lambda_{1,2} = \pm i \sqrt{f_{1v_{\mathrm{m}}} f_{2w} - f_{1w} f_{2v_{\mathrm{m}}}}. \tag{18}$$

These eigenvalues correspond to an oscillatory solution, i.e., spike trains, with a frequency given by

$$\begin{aligned} \omega &= \sqrt{f_{1v_{\mathrm{m}}} f_{2w} - f_{1w} f_{2v_{\mathrm{m}}}} \\ &= \sqrt{\frac{\varepsilon_2}{\varepsilon_1 c_{\mathrm{m}}}} \sqrt{-\gamma q(v_{\mathrm{m}}^*, v_1, v_2, v_3, k) + \beta}. \end{aligned} \tag{19}$$

According to Eq. (7), v_{m}^* in the above equation is a function of v_1, v_2, v_3, k, β, δ, γ, ν. Hence, the frequency can be represented as

$$\omega = \sqrt{\frac{\varepsilon_2}{\varepsilon_1 c_{\mathrm{m}}}} g(v_1, v_2, v_3, k, \beta, \delta, \gamma, \nu), \tag{20}$$

where $g(v_1, v_2, v_3, k, \beta, \delta, \gamma, \nu) = \sqrt{-\gamma q(v_{\mathrm{m}}^*, v_1, v_2, v_3, k) + \beta}$, which is a function of v_1,

v_2, v_3, k, β, δ, γ, ν.

References

1. Hale JK, Buttanri H, Kocak H. Dynamics and Bifurcations. Texts in Applied Mathematics. Springer New York; 1996.
